# Obesity-associated, but not obesity-independent, tumors respond to insulin by increasing mitochondrial glucose oxidation

**DOI:** 10.1371/journal.pone.0218126

**Published:** 2019-06-12

**Authors:** Aviva Rabin-Court, Marcos R. Rodrigues, Xian-Man Zhang, Rachel J. Perry

**Affiliations:** 1 Department of Internal Medicine, Yale University School of Medicine, New Haven, Connecticut, United States of America; 2 Department of Cellular & Molecular Physiology, Yale University School of Medicine, New Haven, Connecticut, United States of America; University of South Alabama, UNITED STATES

## Abstract

Obesity is associated with increased incidence and worse prognosis of more than one dozen tumor types; however, the molecular mechanisms for this association remain under debate. We hypothesized that insulin, which is elevated in obesity-driven insulin resistance, would increase tumor glucose oxidation in obesity-associated tumors. To test this hypothesis, we applied and validated a stable isotope method to measure the ratio of pyruvate dehydrogenase flux to citrate synthase flux (V_PDH_/V_CS_, i.e. the percent of total mitochondrial oxidation fueled by glucose) in tumor cells. Using this method, we found that three tumor cell lines associated with obesity (colon cancer [MC38], breast cancer [4T1], and prostate cancer [TRAMP-C3] cells) increase V_PDH_/V_CS_ in response to physiologic concentrations of insulin. In contrast, three tumor cell lines that are not associated with obesity (melanoma [YUMM1.7], B cell lymphoma [BCL1 clone 5B1b], and small cell lung cancer [NCI-H69] cells) exhibited no oxidative response to insulin. The observed increase in glucose oxidation in response to insulin correlated with a dose-dependent increase in cell division in obesity-associated tumor cell lines when grown in insulin, whereas no alteration in cell division was seen in tumor types not associated with obesity. These data reveal that a shift in substrate preference in the setting of physiologic insulin may comprise a metabolic signature of obesity-associated tumors that differs from that of those not associated with obesity.

## Introduction

Obesity is well-known to increase the prevalence and mortality of more than one dozen tumor types. In spite of the prevalence of obesity and its privileged place in public health discourse, the metabolic and molecular mechanisms underpinning the relationship between obesity and cancer remain contentious. Hyperinsulinemia has emerged as a focal point of research on obesity-related tumors, with increased plasma insulin concentrations independently predicting increased risk and mortality in prostate [1, 2], colon [3–7], breast [8–13], endometrial [12, 14, 15], and pancreatic cancer [16, 17], as well as several other tumor types. The idea that hyperinsulinemia may promote cancer risk is bolstered by the fact that biguanides such as metformin and phenformin, the most commonly prescribed class of diabetes drug worldwide, slow tumor growth associated with reductions in plasma insulin concentrations [18–30], although this class of agents has also shown efficacy *in vivo* independent of changes in plasma insulin concentrations in a minority of studies [31–33]. We recently showed that both metformin and a novel insulin sensitizer, a controlled-release mitochondrial protonophore, slows tumor growth in two models of colon cancer, and that the tumor-slowing effects of both agents were dependent on reversal of hyperinsulinemia [21], demonstrating a causative role for hyperinsulinemia in these mouse models.

While the association between hyperinsulinemia and obesity-related cancer progression is well established, the mechanisms by which hyperinsulinemia may promote tumor growth remain under debate. High doses of dichloroacetate, an indirect activator of pyruvate dehydrogenase and therefore of mitochondrial glucose oxidation, were shown to inhibit proliferation of colorectal cancer cells, particularly under hypoxic conditions [34]; however because these studies were performed in unphysiologic media containing glucose but without pyruvate, lactate, amino acids, or fatty acids, it is difficult to draw strong conclusions regarding the impact of a shift in substrate utilization from glycolytic to oxidative metabolism on tumor cell division under physiologic conditions. To that end, we show here that insulin increases mitochondrial glucose oxidation and augments cell division in cells from obesity-associated tumors, while obesity-independent cell lines show no alteration of substrate preference. These data break with the conventional stance that glucose oxidation is constitutively high in cancer cells, revealing a shift in substrate preference which may comprise a metabolic signature of obesity-related tumors.

## Materials and methods

### Cells

MC38 cells (ENH204) were obtained from Kerafast and YUMM1.7 (CRL-3362), TRAMP-C3 (CRL-2732), BCL1 clone 5B1b (TIB-197), 4T1 (CRL-2539), NCI-H69 (HTB-119), HCT 116 (CCL-247), DLD-1 (CCL-221), B16-F10 (CRL-6475), and COLO 829 (CRL-1974) cells from ATCC. All cells were cultured in the manufacturer’s recommended media, supplemented with penicillin/streptomycin, and were trypsinized and split 2–3 times weekly. Cells were plated in 6 well plates (5x10^5^ cells per well) one day prior to each *in vitro* experiment, and on the day of the experiment were washed twice with warmed PBS prior to the study. For the cell division studies, two insulin doses were chosen: 0.1 nM (the approximate plasma insulin concentration previously measured *in vivo* in overnight fasted rodents [30, 35, 36] and utilized in *in vitro* tumor studies [37–40]) and 100 nM (a dose previously used extensively in *in vitro* studies to assess the impact of insulin on tumor cells [38, 41–45]). Cells were plated in 6 well plates (1x10^5^ cells per well), incubated in the manufacturer’s recommended media with or without insulin (0.1 or 100 μM), dichloroacetate (25 mM in 0.1% DMSO), or 6,8-bis(benzylthio)octanoic acid (1 μM in 0.1% DMSO), and counted by a blinded investigator three days later. These data are presented normalized to controls (without insulin/6,8-bis(benzylthio)octanoic acid) from the same cell line.

### *In vitro* ^13^C labeling studies

The base media used for the ^13^C labeling studies was Dulbecco’s Modified Eagle’s Medium (DMEM) containing 5 mM [^13^C_6_] glucose, nonessential amino acids, 2 mM glutamine, 1 mM lactate, 1 mM palmitate, and 0.1 mM β-OHB. In certain cases, insulin (100 nM), etomoxir (10 μM in 0.1% DMSO), dichloroacetate (25 mM in 0.1% DMSO), or 6,8-bis(benzylthio)octanoic acid (1 μM in 0.1% DMSO) was added, with 0.1% DMSO added to the media in the control wells. Cells were incubated for 2 hours in this media, after which the media was aspirated and cells were washed three times with warmed PBS, after which they were quenched with ice-cold 50% methanol, scraped from the cell plate, and frozen at -80°C pending further analysis.

### Measurement of glucose uptake and oxidation

Glucose uptake was measured by incubating 10^5^ cells per replicate in DMEM containing 5 mM glucose, nonessential amino acids, 2 mM glutamine, 1 mM lactate, 1 mM palmitate, and 0.1 mM β-OHB, and [1-^14^C] 2-deoxyglucose (PerkinElmer) (0.5 μCi per replicate) for 120 min, after which cells were washed three times in PBS, scraped, and collected into scintillation vials. The ^14^C specific activity was quantified using a scintillation counter, and glucose uptake was calculated by assuming a constant isotopically labeled precursor and a constant rate of glucose uptake over the 120 min incubation period.

V_PDH_/V_CS_ was measured in cells incubated in [^13^C_6_] glucose as VPDHVCS=[4,5-C132]glutamate[C133]alanine [35, 46]. Briefly, this method employs measurement of [4,5-^13^C_2_] glutamate as equivalent to [^13^C_2_] acetyl-CoA, the product of PDH (S1 Fig), whereas [^13^C_3_] alanine serves as a reciprocal pool for [^13^C_3_] pyruvate, the latter of which is found at lower concentrations and is much more labile, rendering it difficult to reliably measure under these conditions. Cell samples quenched in 50% methanol were prepared for LC-MS/MS analysis of [4,5-^13^C_2_] glutamate enrichment and GC/MS analysis of [^13^C_3_] alanine enrichment as we have described [35].

Absolute rates of glucose oxidation were determined in MC38 and YUMM1.7 cells by incubating 5x10^5^ cells in sealed flasks for 30 min in DMEM culture media (5 mM glucose, nonessential amino acids, 2 mM glutamine, 1 mM lactate, 1 mM palmitate, and 0.1 mM β-OHB) containing 0.2 μCi [^14^C_6_] glucose. The [^14^CO_2_] produced was trapped on Whatman paper in a holder suspending it in the air above the cells and, after 30 min of incubation in ^14^C media, the ^14^C activity was determined using a scintillation counter.

### Assessment of lactate production

To measure lactate production, 10^5^ cells were washed three times with warmed PBS and placed in DMEM containing 5 mM glucose, nonessential amino acids, 2 mM glutamine, 1 mM palmitate, and 1 mM β-OHB, but without lactate or pyruvate. After 120 min, the media was collected and spiked with ^13^C_3_ lactate (3 ng). The concentration of lactate in the media was measured by determining the ratio of ^13^C_3_ to ^12^C lactate by GC/MS using the same method as we have previously published to examine alanine concentrations/enrichment in plasma and tissues [35]. The rate of net lactate production was calculated by assuming linear accumulation of lactate in the media over time, and assuming an unchanged concentration of lactate in the cells.

### Measurement of V_β-OHB-ox_/V_CS_

V_β-OHB-ox_/V_CS_ was measured by incubating cells in DMEM containing 1 mM [^13^C_4_] β-OHB, 5 mM glucose, nonessential amino acids, 2 mM glutamine, 1 mM lactate, and 1 mM palmitate. Using this tracer, V_β-OHB-ox_/V_CS_ is given as Vβ-OHB-oxVCS=[4,5-C132]glutamate[C134]β-OHB.

Cell samples quenched in 50% methanol were prepared for LC-MS/MS analysis of [4,5-^13^C_2_] glutamate enrichment and GC/MS analysis of [^13^C_3_] alanine enrichment as described above and in our previous report [35]; [^13^C_4_] β-OHB enrichment was measured using the same GC/MS method as was used for [^13^C_3_] alanine.

### Assessment of insulin receptor expression and phosphorylation

Insulin receptor expression and pTyr^1162^ phosphorylation was measured by Western blot using antibodies from Cell Signaling (catalog numbers 3025 and 3918, respectively) and normalized to beta-actin expression (Cell Signaling #9457).

### Statistical analysis

Statistical analysis was performed using Prism 7.0. Groups were compared by the 2-tailed unpaired Student’s t-test (for comparisons of two groups) or by ANOVA with Bonferroni’s multiple comparisons test (for comparisons of three or more groups) after verifying that the data met the assumptions of the statistical test employed.

## Results

### Insulin activates the insulin receptor in all tumor cell lines

**Fig 1:**
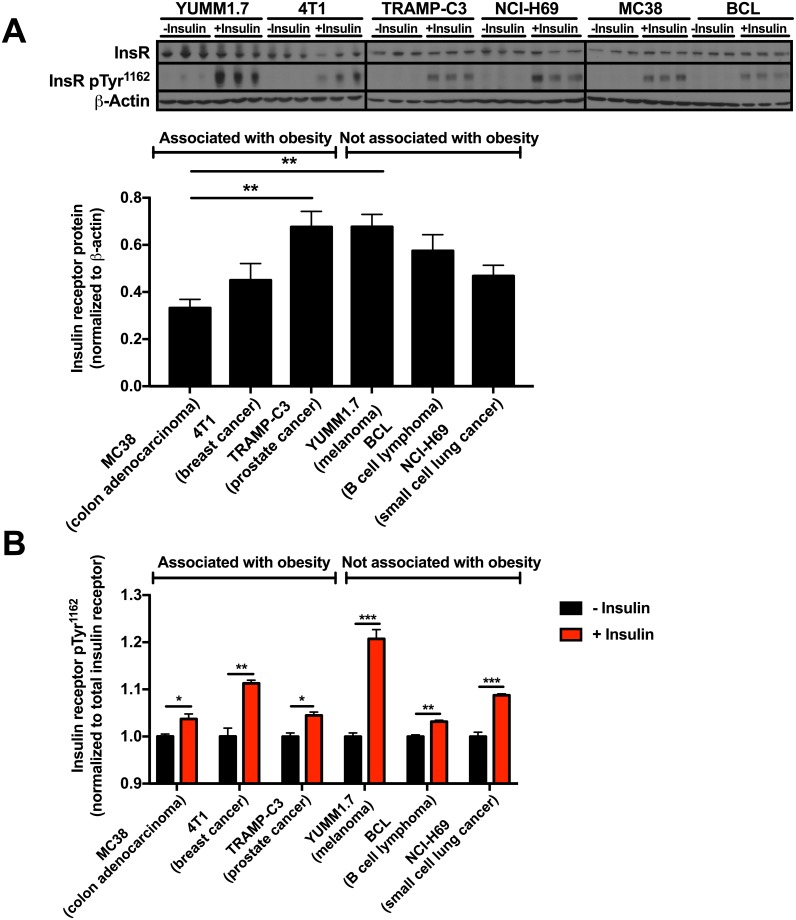
Insulin receptor expression and insulin-stimulated insulin receptor phosphorylation do not correlate with tumor cell lines’ association with obesity. (A) Insulin receptor expression. The data are the mean ± S.E.M. of six replicates from each cell line, including cells with and without insulin. **P<0.01 by ANOVA with Bonferroni’s multiple comparisons test. (B) Insulin receptor Tyr^1162^ phosphorylation. The data are presented as fold change from baseline, representing cells from the same cell line without insulin. *P<0.05, **P<0.01, ***P<0.001 by the 2-tailed unpaired Student’s t-test. Data are the mean ± S.E.M. of n = 3 per condition.

All cell lines employed in this study robustly expressed the insulin receptor, and differences in insulin receptor expression between cell lines did not correlate with obesity association or lack thereof (Fig 1A). Insulin activated the insulin receptor, as indicated by increased insulin receptor Tyr^1162^ phosphorylation [47] in all cell lines regardless of their correlation, or lack thereof, with obesity (Fig 1B).

### Insulin increases glucose oxidation in obesity-related, but not obesity-independent, cancer cell lines

**Fig 2:**
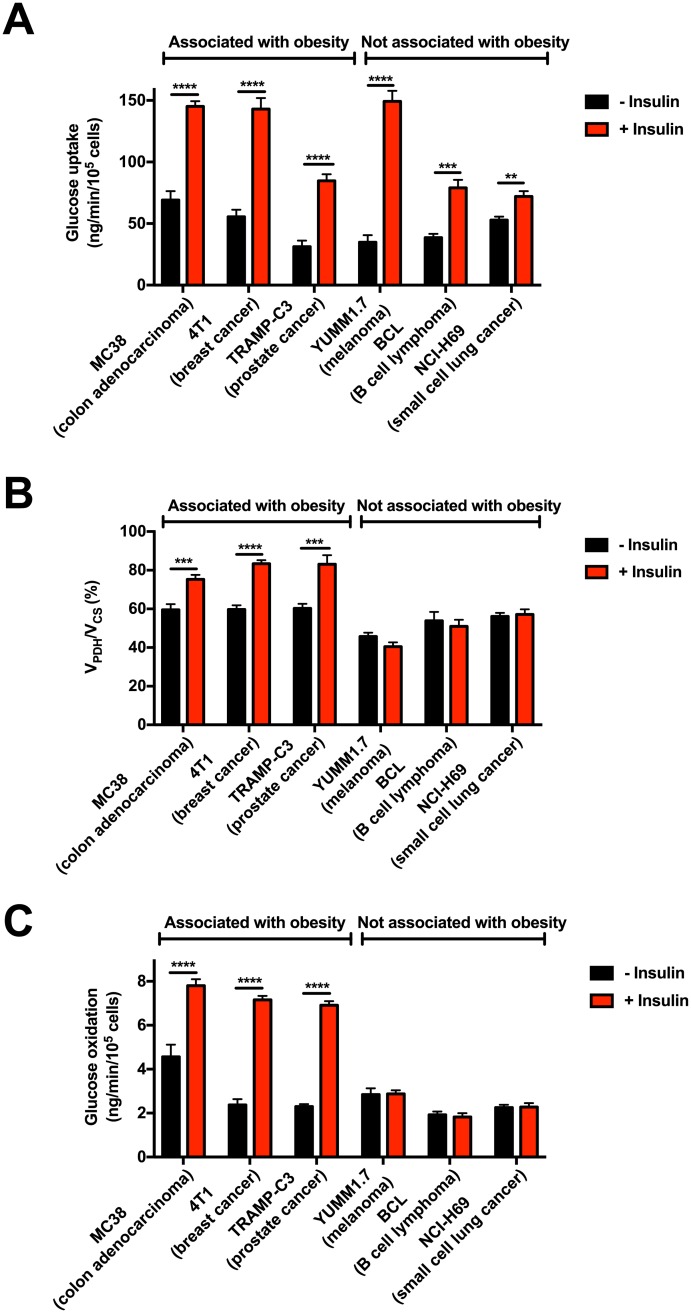
Tumor types associated with obesity, but not those not associated with obesity, increase mitochondrial glucose oxidation in the presence of insulin. (A) Glucose uptake. (B) V_PDH_/V_CS_. (C) Absolute rates of glucose oxidation. In all panels, data are the mean ± S.E.M. of n = 5–9 replicates per condition, with cells from the same line ± insulin compared using the 2-tailed unpaired Student’s t-test.

To identify differences in tumor metabolism in obesity-related cancers, as opposed to obesity-independent cancers, we investigated substrate preference in three cell lines from obesity-associated cancer types, each of which exhibits accelerated tumor growth associated with obesity: MC38, a colon adenocarcinoma [19, 30, 48–50], 4TI, a triple-negative breast cancer [51, 52], and TRAMP-C3, a prostate adenocarcinoma [53]. These cell lines were compared with three cancer cell lines from tumor types not associated with obesity: YUMM1.7, melanoma; BCL1 clone 5B1b, B-cell lymphoma; and NCI-H69, small-cell lung cancer. Insulin increased glucose uptake in all cell lines, independent of their association, or lack thereof, with obesity (Fig 2A). However, the ratio of pyruvate dehydrogenase flux to citrate synthase flux (V_PDH_/V_CS_, i.e. the percent of total mitochondrial oxidation fueled by glucose oxidation, S1 Fig) increased in all obesity-associated tumor types in the presence of insulin (100 nM); in contrast, all three obesity-independent cell lines showed no increase in glucose oxidation in response to insulin (Fig 2B). Instead, YUMM1.7 and BCL cells increased and NCI-H69 tended to increase lactate production after insulin treatment (S2A Fig), indicating that glucose taken up in response to insulin is diverted into lactate production if it is not shuttled into mitochondrial oxidation in response to insulin. Finally we confirmed the differential ability of insulin to stimulate glucose oxidation in obesity-associated versus obesity-independent tumor cells in studies of absolute glucose oxidation measured by trapping ^14^CO_2_ generated by oxidizing ^14^C_6_ glucose (Fig 2C): incubation with insulin doubles colon cancer cell glucose oxidation, while melanoma cells show no change in glucose oxidation rates when insulin is added to media.

### Utilization of glutamine and ketones is minimal in MC38 and YUMM1.7 cells

**Fig 3:**
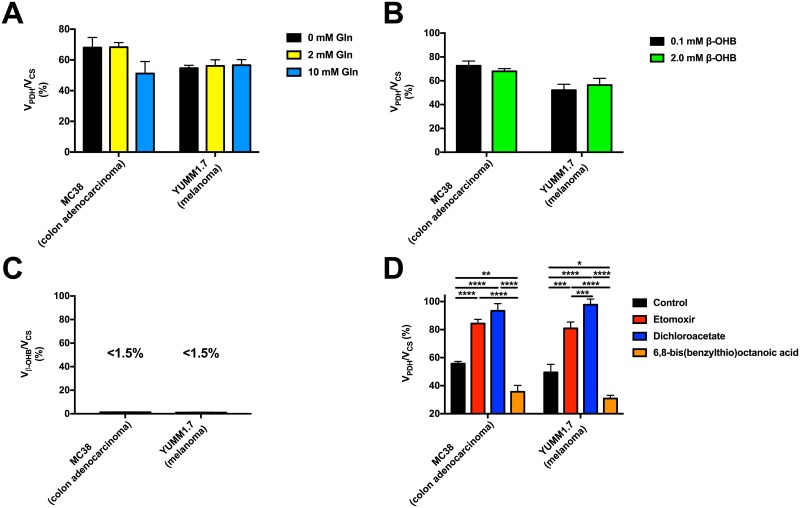
The stable isotope method applied in this study is sensitive to detect the expected differences in V_PDH_/V_CS_ with physiologic alterations in these fluxes, and is not affected by physiologically relevant glutamine or ketone concentrations. (A) Glutamine in the physiologic range (0–10 mM) does not significantly affect the measured V_PDH_/V_CS_ in MC38 or YUMM1.7 cells. n = 4 replicates per condition, with comparisons by ANOVA with Bonferroni’s multiple comparisons test. (B) β-OHB in the physiologic range (0–2 mM) does not significantly alter the measured V_PDH_/V_CS_ in MC38 or YUMM1.7 cells. Conditions were compared by the 2-tailed unpaired Student’s t-test. In panels (B)-(D), n = 6–9 replicates per condition. (C) V_β-OHB-ox_/V_CS_ is minimal in MC38 and YUMM1.7 cells. (D) V_PDH_/V_CS_ is increased with inhibition of fatty acid oxidation (etomoxir) or stimulation of PDH (dichloroacetate), and decreased with inhibition of PDH (6,8-bis(benzylthio)octanoic acid). Groups were compared by ANOVA with Bonferroni’s multiple comparisons test. In all panels, data are the mean ± S.E.M.

To ensure that physiologic levels of glutamine do not significantly affect our measurements of V_PDH_/V_CS_ by dilution of glutamate, MC38 and YUMM1.7 cells were incubated in increasing levels of glutamine. No significant change in V_PDH_/V_CS_ was found between within the physiologic range of glutamine (Fig 3A), indicating that glutamine utilization has minimal impact on the measured V_PDH_/V_CS_ ratio.

Next, to determine the potential contribution of ketone oxidation to tumor metabolism, we incubated both MC38 and YUMM1.7 cells in low and high concentrations of β-hydroxybutyrate (β-OHB) and found that increased β-OHB concentrations have no impact upon V_PDH_/V_CS_ (Fig 3B). To confirm this, we measured the contribution of ketone oxidation to total mitochondrial oxidation, V_β-OHB_/V_CS_ (S1 Fig), and found that ketone oxidation is minimal in both MC38 and YUMM1.7 cells *in vitro* (Fig 3C). These findings indicate a central role for glucose oxidation and, to a lesser extent, fatty acid oxidation in cancer metabolism, in the absence of a key role for ketone oxidation.

### V_PDH_/V_CS_ is altered as expected with pharmacologic manipulation of fatty acid and glucose oxidation

To validate the sensitivity of our method to detect differences in V_PDH_/V_CS_, we employed three pharmacologic agents to alter substrate oxidation: etomoxir, an inhibitor of fatty acid oxidation; dichloroacetate, a PDH activator, and 6,8-bis(benzylthio)octanoic acid, a PDH inhibitor (Fig 3D). Inhibition of fatty acid oxidation led to a relative increase in V_PDH_/V_CS_ in both MC38 and YUMM1.7 cells, reflecting a decrease in fatty acid oxidation relative to citrate synthase flux. As expected, activation of PDH with dichloroacetate increased the measured V_PDH_/V_CS_ ratio in both cell lines, whereas PDH inhibition with 6,8-bis(benzylthio)octanoic acid reduced this ratio. The response of MC38 and YUMM1.7 cells to each small molecule glucose or fat oxidation modulator was similar, demonstrating that the differing responses of the cell lines to insulin was not attributable to inherent alterations in mitochondrial function. Taken together, these data demonstrate that our stable isotope method has the necessary sensitivity to detect expected differences in glucose or fatty acid oxidation under these conditions *in vitro*.

### Insulin increases cell division in obesity-associated, but not obesity-independent, tumor cell types

**Fig 4:**
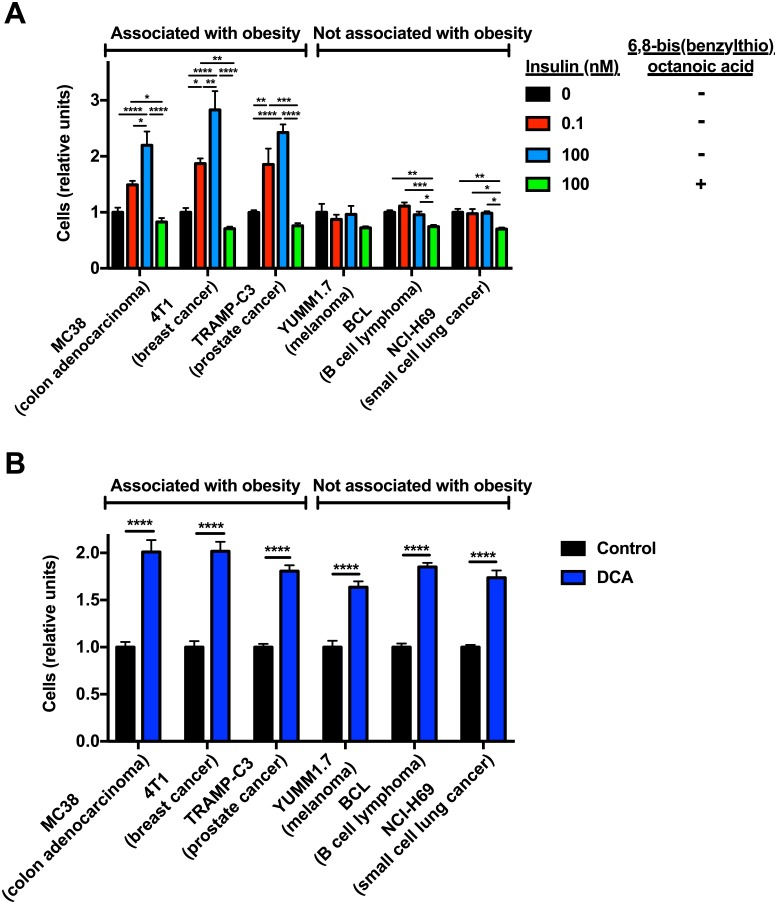
PDH activity promotes tumor growth in both obesity-associated and obesity-independent tumor cell lines. (A) Cell division in tumor cell lines incubated with or without insulin, with or without a PDH inhibitor. (B) Cell division in tumor cell lines incubated with or without the PDH activator dichloroacetate. In both panels, n = 6 replicates per condition, normalized to controls from the same cell line. *P<0.05, **P<0.01, ***P<0.001, ****P<0.0001 by ANOVA with Bonferroni’s multiple comparisons test (panel (A)) or by the 2-tailed unpaired Student’s t-test (panel (B)). Data are the mean± S.E.M.

To determine whether the observed increases in V_PDH_/V_CS_ in response to insulin affected cell division, we incubated all cell types in insulin (0.1 and 100 nM) and found that insulin increased rates of cell division in all three obesity-associated cell lines, but in none of the cell lines from tumors not associated with obesity (Fig 4A). By examining two additional colon cancer cell lines and two additional melanoma cell lines, we then confirmed that the insulin response is conserved across tumor types: all three colon cancer cell lines increased cell division in response to incubation in 0.1 nM insulin, whereas none of the melanoma cell lines exhibited this response (S2B Fig). However, inhibition of PDH using 6,8-bis(benzylthio)octanoic acid reduced cell division in both obesity-associated and -independent cell lines. Conversely, activation of PDH with DCA promoted tumor cell division in all cell lines (Fig 4B). Taken together, these data demonstrate that PDH-mediated glucose oxidation, which increases in response to insulin in obesity-associated but not obesity-independent tumor cell lines, drives cell division in these six tumor models, at least in *in vitro* culture.

## Discussion

Hyperinsulinemia has been discussed as a potential mediator of obesity-related cancer growth: activating mutations in the PI3K/Akt pathway are common (10–30% incidence) and confer a poorer prognosis in humans with colon cancer [54, 55], breast cancer [56, 57], and prostate cancer [58, 59]. Activating mutations in the PI3K/Akt pathway have also been observed–albeit at a lower incidence (2–9%)–in melanoma [60, 61] and small cell lung cancer [62, 63], although, to our knowledge, they have not been observed in B cell lymphoma [64, 65]. Mutations in this pathway do not divide as clearly along obesity-associated versus independent lines in the cell lines examined in this study: whereas mutations in this pathway have been described in 4T1 [66] and NCI-H69 cells [67], they have not been described in the other obesity-associated or -independent cell lines studied here. Evidence for a critical role for insulin in directly promoting tumor cell division is provided by data showing that while insulin promotes tumor cell division *in vivo* [30, 68–71], pharmacologic agents which reverse hyperinsulinemia slow tumor growth unless exogenous insulin replacement is provided [30]. However, the mechanisms undergirding insulin’s effect upon certain tumors have remained unclear, in part due to a lack of methods to assess tumor substrate preference and the impact of variations in hormones and substrates on tumor fuel preference. To that end, we adapted a stable isotope method which we have previously applied *in vivo* for tissue-specific measurements of glucose oxidation via pyruvate dehydrogenase relative to total citrate synthase flux [30, 35]. Validation studies demonstrate the sensitivity of this method to alterations in glucose and fatty acid oxidation, and further demonstrate that glucose oxidation is not maximized under these conditions; therefore the lack of an oxidative response to insulin in obesity-independent cell lines does not reflect an inherent limitation in mitochondrial glucose utilization in these cells (Fig 3D).

Somewhat unexpectedly, we found that V_PDH_/V_CS_ measured as the ratio of [4,5-^13^C_2_] glutamate/[^13^C_3_] alanine was unaltered by the presence or absence of physiologic (2 and 10 mM) concentrations of glutamine. These data stand in contrast to studies, which have previously been extensively reviewed [72–74], indicating that glutamine metabolism plays a critical role in cancer cell metabolism. It is possible that glutamine may be used preferentially for nucleotide synthesis rather than mitochondrial oxidation when the preferred glycolytic metabolites (glucose, lactate, pyruvate) are available, and may be required for oxidative metabolism only when substrate supply is limiting due to inadequate vascularization. Future tracer studies utilizing ^13^C glutamate as a surrogate for ^13^C acetyl-CoA, the immediate product of PDH, would need to confirm a minimal contribution of glutamine to oxidative metabolism; otherwise, [^13^C_2_] acetyl-CoA would need to replace [4,5-^13^C_2_] glutamate in the numerator of this ratio.

Using our stable isotope method, which we have now validated in multiple tumor cell types, we show here that three obesity-associated tumor cell lines respond to insulin by increasing glucose oxidation relative to total mitochondrial oxidation. This effect suggests that the mechanism by which obesity-related tumors capitalize upon their hyperinsulinemic environment is conserved across tumor types. These data were validated by use of an entirely independent method, trapping [^14^CO_2_] generated by the oxidation of [^14^C] glucose, which showed that rates of absolute glucose oxidation increase similarly to the change in V_PDH_/V_CS_ upon insulin stimulation in MC38 colon cancer, 4T1 breast cancer, and TRAMPC3 prostate cancer cells, but are unaltered by insulin in YUMM1.7 melanoma, BCL B cell lymphoma, and NCI-H69 small cell lung cancer cells. This method of measuring absolute rates of glucose oxidation, which has been applied extensively in various cell types including neurons and astroglia [75, 76], oocytes [77], hepatocytes [78], intestine [79], and heart [80, 81], as well as tumor cells [82, 83], relies upon a set of assumptions that differ significantly from those of the V_PDH_/V_CS_ method; the similar impact of insulin upon absolute glucose oxidation and V_PDH_/V_CS_ lends further credence to the latter method. In contrast to the three obesity-associated tumor types, melanoma, B cell lymphoma, and small cell lung cancer cell lines showed no mitochondrial oxidative response to insulin. The oxidative response to insulin did not correlate with insulin receptor expression or activation: insulin receptor expression did not differ between cell types. However while insulin-stimulated glucose uptake correlated with insulin receptor activation, neither parameter was associated with the oxidative response to insulin: insulin increased both insulin receptor phosphorylation (Fig 1B) and glucose uptake (Fig 2A) in all cell lines to varying degrees, insulin promoted glucose oxidation only in obesity-associated tumors (Fig 2B and 2C). However, insulin did not decrease lactate production in obesity-associated cells, consistent with the fact that while the insulin-stimulated increase in glucose uptake outpaced insulin-stimulated increases in mitochondrial oxidation in all cells, excess glucose may be used for a variety of synthetic pathways (including glycogen synthesis and/or the energetic requirements of cell division) as well as lactate production. These increases in tumor glucose oxidation correlated with accelerated cell division in response to insulin: whereas there was no difference in cell division whether or not melanoma, small cell lung cancer, or B cell lymphoma cells were incubated in insulin, the three obesity-associated tumor types each showed a dose-dependent increase in cell division with insulin (Fig 4A, S2B Fig). Importantly, due to their differing doubling times, the magnitude of the impact of insulin on cell division should not be compared across different tumor types; however, this study does show that insulin promotes cell division in each of five obesity-associated tumor types, but not in any of the five obesity-independent tumor cell lines studied. However, activation of PDH increased and inhibition of PDH inhibited cell division in all six cell lines tested, regardless of their association, or lack thereof, with obesity (Fig 4B). These data indicate that the idea that a switch from glycolytic to oxidative glucose metabolism will slow tumor cell proliferation may be an oversimplification and that under conditions in which substrate limitation is not present, oxidative metabolism may actually promote tumor cell division. Taken together, this study underscores the possibility that upregulated glucose oxidation and increased V_PDH_/V_CS_ under conditions of hyperinsulinemia may constitute a metabolic signature of obesity-related cancer and recommends further studies to explore the link between hyperinsulinemia, increased glucose uptake, and tumor cell division *in vivo*.

## Supporting information

S1 Fig Tracer labeling scheme. (A) Isotopomers generated on the first turn (solid red circles) and second turn (dashed red circles) of the tricarboxylic acid cycle during incubation in [^13^C_6_] glucose. V_PDH_, pyruvate dehydrogenase flux. V_PC_, pyruvate carboxylase flux. V_CS_, citrate synthase flux. OAA, oxaloacetate. α-KG, alpha-ketoglutarate. (B) Isotopomers generated on the first turn (solid red circles) and second turn (dashed red circles) of the tricarboxylic acid cycle during incubation in [^13^C_4_] β-hydroxybutyrate. V_β-OHB-ox_, ketone (β-hydroxybutyrate) oxidation.
(PDF)

S2 Fig Insulin promotes glucose oxidation but not lactate production and cell division in obesity-associated associated but not obesity-independent tumor cell lines. (A) Rate of lactate production. (B) Impact of insulin (0.1 nM) on cell division. Data for MC38 and YUMM1.7 cells are duplicated from Fig 4A. In both panels, n = 6 replicates per condition. *P<0.05, **P<0.01, ****P<0.0001 by the 2-tailed unpaired Student’s t-test.
(PDF)
