# Supplementary figures and images for: Obesity-associated, but not obesity-independent, tumors respond to insulin by increasing mitochondrial glucose oxidation
Source: PLoS One. 2019 Jun 12;14(6):e0218126. doi: 10.1371/journal.pone.0218126 (PMC6561592; doi:10.1371/journal.pone.0218126)

# S1 Fig

A

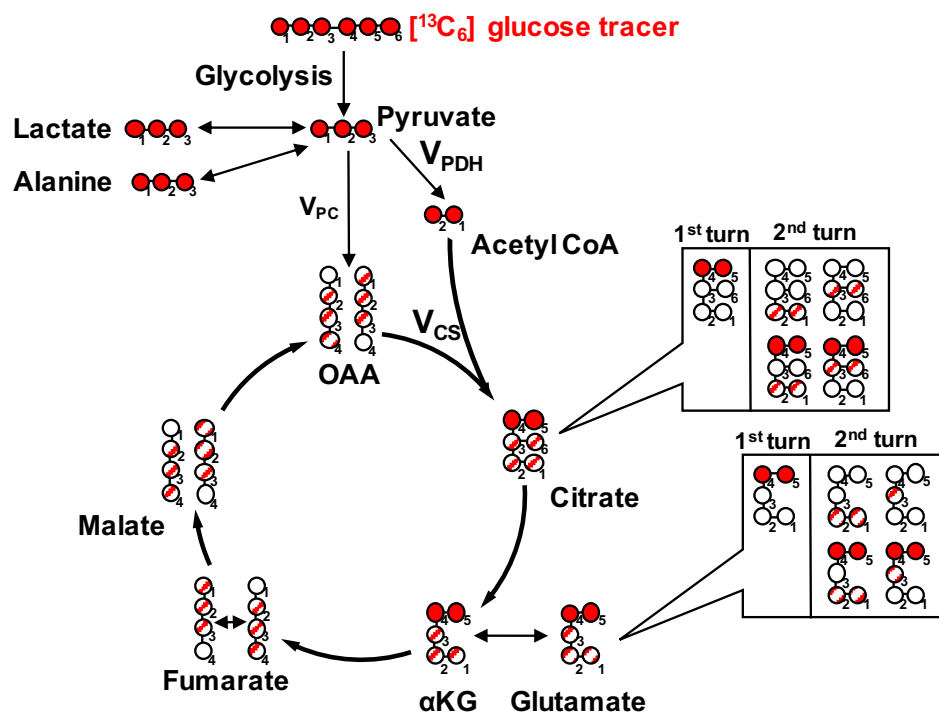

B

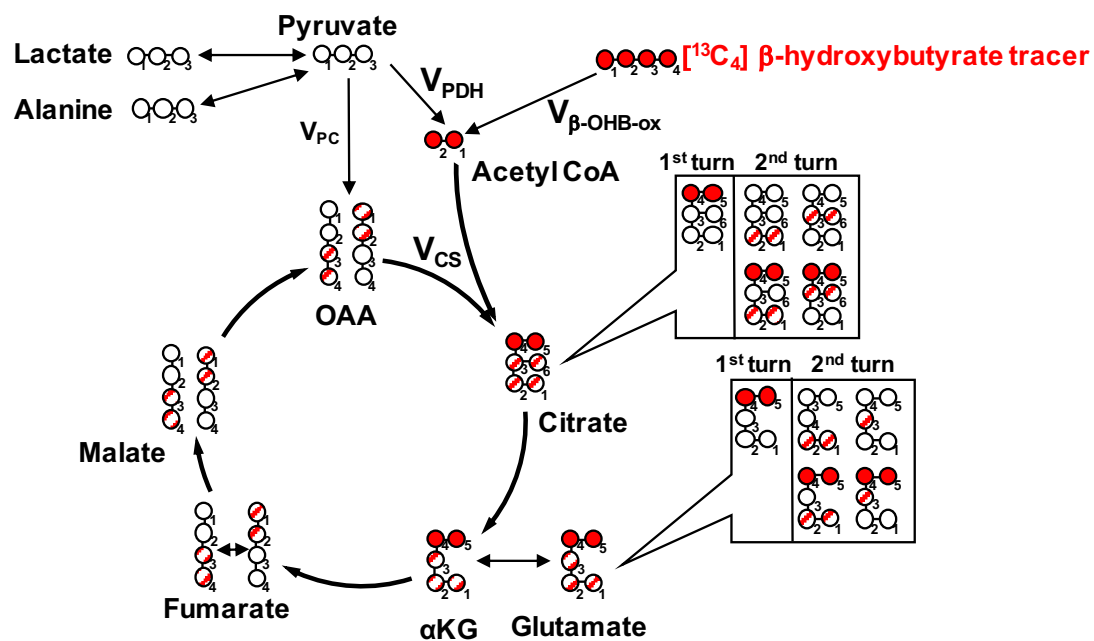

Supplement: S1 Fig — (A) Isotopomers generated on the first turn (solid red circles) and second turn (dashed red circles) of the tricarboxylic acid cycle during incubation in [13C6] glucose. VPDH, pyruvate dehydrogenase flux. VPC, pyruvate carboxylase flux. VCS, citrate synthase flux. OAA, oxaloacetate. α-KG, alpha-ketoglutarate. (B) Isotopomers generated on the first turn (solid red circles) and second turn (dashed red circles) of the tricarboxylic acid cycle during incubation in [13C4] β-hydroxybutyrate. Vβ-OHB-ox, ketone (β-hydroxybutyrate) oxidation. (PDF) [file pone.0218126.s001.pdf]

# S2 Fig

A

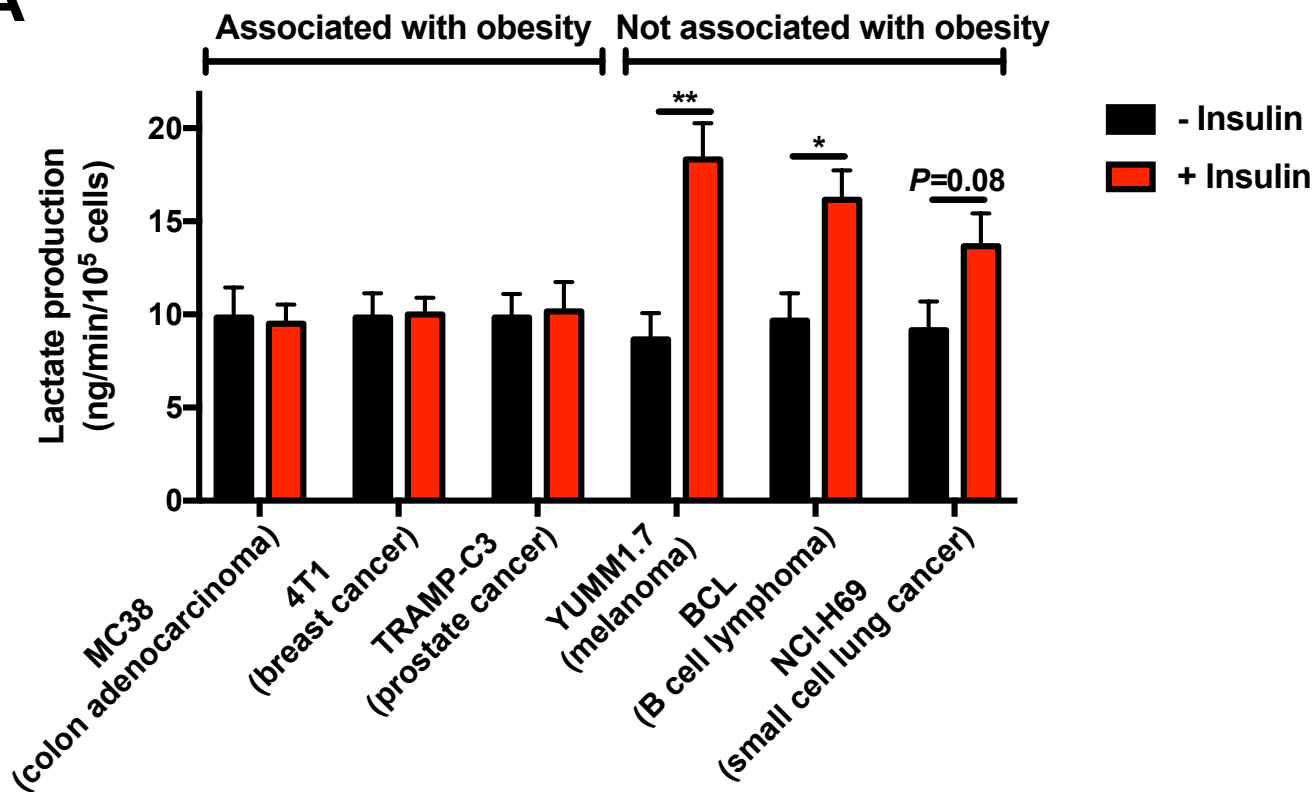

B

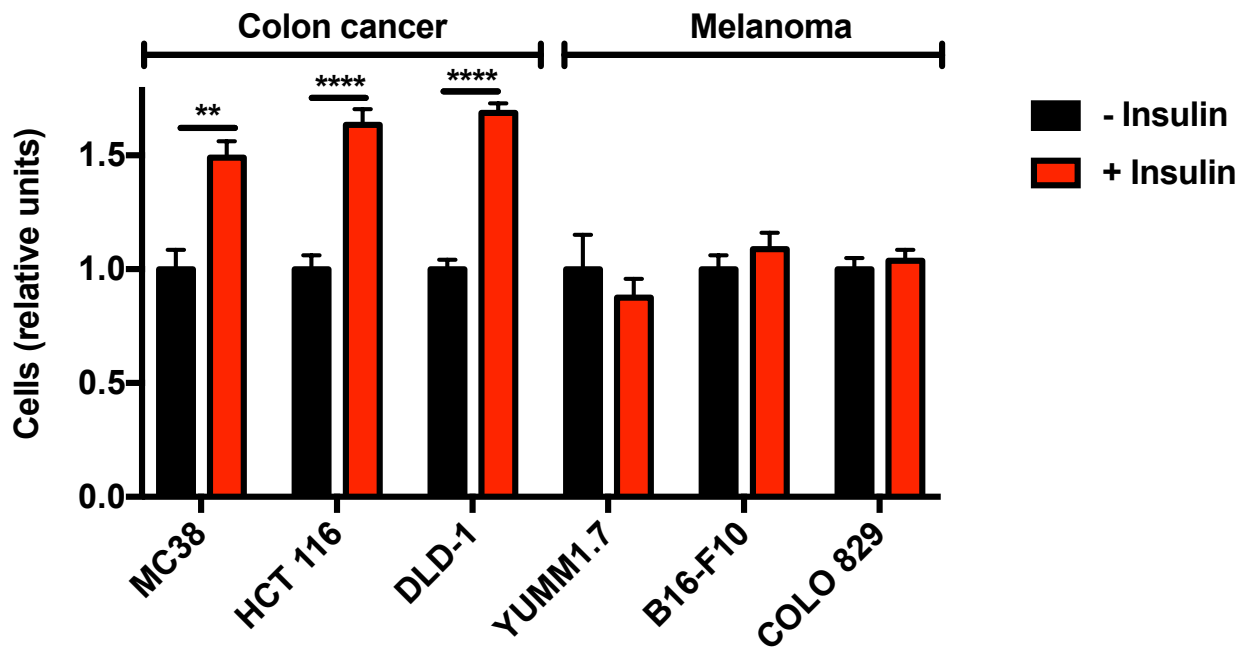

Supplement: S2 Fig — (A) Rate of lactate production. (B) Impact of insulin (0.1 nM) on cell division. Data for MC38 and YUMM1.7 cells are duplicated from Fig 4A. In both panels, n = 6 replicates per condition. *P<0.05, **P<0.01, ****P<0.0001 by the 2-tailed unpaired Student’s t-test. (PDF) [file pone.0218126.s002.pdf]
